# Carbon Dot-Enhanced Doxorubicin Liposomes: A Dual-Functional Nanoplatform for Cancer Therapy

**DOI:** 10.3390/ijms26157535

**Published:** 2025-08-04

**Authors:** Corina-Lenuta Logigan, Cristian Peptu, Corneliu S. Stan, Gabriel Luta, Crina Elena Tiron, Mariana Pinteala, Aleksander Foryś, Bogdan Simionescu, Constanta Ibanescu, Adrian Tiron, Catalina A. Peptu

**Affiliations:** 1 Department of Natural and Synthetic Polymers, Faculty of Chemical Engineering and Environmental Protection, “Gheorghe Asachi” Technical University of Iasi, 700050 Iasi, Romania; savincorina@yahoo.com (C.-L.L.); corneliu-sergiu.stan@academic.tuiasi.ro (C.S.S.); bcsimion@icmpp.ro (B.S.); constantaib@yahoo.com (C.I.); 2 “Petru Poni” Institute of Macromolecular Chemistry, 700487 Iasi, Romania; cristian.peptu@icmpp.ro (C.P.); pinteala@icmpp.ro (M.P.); 3 Regional Institute of Oncology, 700483 Iasi, Romania; gabriel.luta@iroiasi.ro (G.L.); transcendctiron@iroiasi.ro (C.E.T.); 4 Centre of Polymer and Carbon Materials of the Polish Academy of Sciences, 41-819 Zabrze, Poland; aforys@cmpw-pan.pl

**Keywords:** liposomes, carbon dots (CDs-NHF), drug delivery, breast cancer, lung cancer, doxorubicin synergy, signal transduction inhibition

## Abstract

Liposomes (LPs) represent one of the most effective nanoscale platforms for drug delivery in cancer therapy due to their favorable pharmacokinetic and various body tissue compatibility profiles. Building on recent findings showing that carbon dots derived from N-hydroxyphthalimide (CDs-NHF) possess intrinsic antitumor activity, herein, we investigate the possibility of preparing complex nano-platforms composed of LPs encapsulating CDs-NHF and/or doxorubicin (DOX) for breast and lung cancer. Various LP formulations were prepared and characterized using Cryo-TEM and Cryo-SEM for morphological analysis, while zeta potential and fluorescence assessments confirmed their stability and optical properties. Cellular effects were evaluated through immunofluorescence microscopy and proliferation assays. LPs-CDs-NHF significantly reduced cancer cell viability at lower concentrations compared to free CDs-NHF, and this effect was further amplified when combined with doxorubicin. Mechanistically, the liposomal formulations downregulated key signaling molecules including pAKT, pmTOR, and pERK, indicating the disruption of cancer-related pathways. These findings suggest that LPs containing CDs-NHF, either alone or in combination with DOX, exhibit synergistic antitumor activity and hold strong promise as multifunctional nanocarriers for future oncological applications.

## 1. Introduction

During the last decade, new, advanced, and promising applications have been developed based on carbon dots (CDs). The CDs are attractive nanomaterials due to their outstanding physicochemical and photoluminescent properties facilitating the synthesis and encapsulation of novel materials, with potential applications in optoelectronics, catalysis, and biomedicine [1,2]. As well, CDs have unique structural, thermal, mechanical, electrical, and optical properties, respectively; facile functionalization, the possibility of entrapment in various polymeric matrices, low toxicity, and biocompatibility, which generate particular interest for different biomedical applications including drug/gene delivery, tissue engineering, bioimaging, respectively; and diagnoses and cancer therapy [2,3].

Within the last years, different types of drug delivery systems targeting breast and lung cancer were developed for imparting the selective delivery of drugs to the desired site [4,5,6,7]. Moreover, there is an increasing interest in the development of nano-theranostically engineered multimodal platforms for cancer treatment. In this context, few studies are focused on the usefulness of a natural/polymeric delivery system capable of integrating CDs’ physicochemical and photoluminescent properties [8].

Liposomes are usually sphere-shaped vesicles with a size range of 10 nm^−1^ μm or greater, consisting of multi-lipid layers surrounding an aqueous core enclosed by phospholipid bilayers of natural or synthetic origin. LPs present a good affinity for cell membranes and can easily bind to cell surfaces and facilitate drug transfer. Liposomal formulations have been extensively investigated as a carrier and a great choice for the delivery of therapeutic agents. Compared with other delivery systems, LPs possess good properties like cell-like membrane structure, biocompatibility, biodegradability, low toxicity, and a hydrophobic, hydrophilic character which facilitates the incorporation and targeted delivery of both hydrophobic and hydrophilic molecules [9,10].

Several approved LPs for the treatment of cancer include Onivyde™, a pegylated liposomal formulation for the treatment of pancreatic cancer; Marqibo^®^, a liposomal formulation based on vincristine liposome for the treatment of blood cancer; Doxil^®^, a liposomal doxorubicin formulation for the treatment of breast cancer; Visudyne^®^, a liposomal formulation containing verteporfin used along with laser light treatment to treat certain serious eye conditions (such as macular degeneration, pathologic myopia, and ocular histoplasmosis); and Depocyt^®^, used for the treatment of acute nonlymphocytic leukemia, meningeal leukemia, refractory leukemia, and lymphomatous meningitis [11].

All the recent advancements in the clinical management of many types of cancers, such as breast, lung, and colorectal cancers, are still challenging due to advanced evolution, escape from screening, and rapidly acquired resistance [12]. Cancer therapy, typically surgery, chemotherapy, and radiotherapy, have several limitations like nonspecific targeting, inducing multi-drug resistance, and excessive toxic effects leading to treatment failure and disease recurrence [13,14,15].

Various signaling pathways, like PI3K/AKT/mTOR and EGF/RAS/RAF/MEK/ERK, are pivotal in oncogenesis, associated with cancer progression and drug resistance in different types of human cancer [16,17,18]. Several drugs targeting those pathways are in clinical trials, mainly in combination with different therapies since monotherapy was not satisfactory [19,20].

One of the most popular anticancer agents, Doxorubicin (DOX), is used for the treatment of different types of cancers like ovarian, breast, prostate, lung, etc. [13]. One of our recent studies demonstrated that the pyrolytic processing of chemicals such as NHF (*N*-hydroxyphthalimide) and NHS (*N*-hydroxysuccinimide) results in CDs with interesting antitumor activities [21].

Nanomedicine progress concerning cancer therapy allows for a more personalized and precise delivery, and combined with other treatments, had a direct effect on local tumor development and outcome [22]. The nanomedicine approach may comprise nanostructured materials between 5 and 200 nm in diameter which carry therapeutic, diagnostic, and theranostic compounds. In oncology, clinically approved nanostructures are molecular drug carriers designed to improve tumor drug delivery which includes polymeric/inorganic nanoparticles, liposomes (LPs), protein–drug conjugates dendrimers, etc. [23,24,25]. Due to the typically leaky and disorganized nature of tumor vasculature, cancer cells can readily escape systemic circulation and infiltrate the surrounding extracellular space. Because of that, nanomaterial drug carriers are preferentially entrapped in tumors and excluded by normal tissues. Also, nanocarrier surfaces can be decorated with diverse structures like polymers, proteins, and antibodies designed to prevent immune detection and improve targeting and uptake in specific cell populations [26,27,28,29].

Embedding CDs in different matrices such as LPs [9], natural/polymeric nanoparticles or capsules [30], and dendrimers [31] has great potential as a delivery system with enhanced anticancer activity of the CDs. Moreover, a theranostic system formed from a liposomal system including CDs or a drug or both can allow the observation of the CDs/drug localization at a particular site (through fluorescence detection), visualization of its bio-distribution, and evaluation of the therapeutic efficacy [32].

In this context, the literature includes only a few studies on liposomal formulations encapsulating CDs. Demir et al. demonstrated in a relatively recent study the use of liposomes for a theranostic purpose as a novel multimodal platform for a CDs, curcumin, and anti-CD44 antibody combination for cancer treatment. They found that the prepared theranostic LPs-loaded anti-CD44 antibody showed an enhanced effect on cancer cells compared to curcumin-loaded and CUR/CD-loaded LPs [33]. Guan et al. [34] succeeded in preparing glycosylated LPs-loaded CDs for the targeted recognition of HepG2 cells. The results showed that obtained Man-CDs-LPs have a dual use and can be used for specific recognition, but also for tracking the interaction between carbohydrates and glycoprotein by the fluorescence images of CDs. Another study concerning CDs-loaded LPs was performed by Ren et al. [35] The developed multifunctional LPs were loaded with a hydrophilic near-infrared CD as a nanocarrier and tracer of the lipophilic anticancer agent cinobufagin. The LPs displayed some advantages such as lysosomes-targeted cellular imaging, tumor-targeted delivery, enhanced photoluminescence emission/imaging, enhanced cellular uptake, and improved anticancer activity.

This study focuses on developing various LPs formulations as multifunctional delivery systems for CDs-NHF and/or DOX. We hypothesize that LPs formulations can enhance the efficacy of CDs-NHF by improving their water solubility and reducing aggregation, which otherwise limits their clinical antitumor potential. Liposomes (LPs) prepared using the reverse-phase evaporation method demonstrated an effective and reliable approach for liposome production. The resulting LPs showed an excellent capacity for encapsulating CDs-NHF/DOX and may be considered as a potential nano-formulation for antitumor applications. Moreover, our previous works [36,37] succeeded in proving that CDs-NHF entrapped in a polymeric matrix presented antitumoral activity. In this investigation, we describe a slightly modified method for preparing various LPs formulations based on Phospholipon G and cholesterol using the reverse-phase evaporation technique. LPs characterization was carried out in terms of size (dynamic light scattering—DLS), morphology visualized via Cryo-Transmission Electron Microscopy (Cryo-TEM), Cryo-Scanning Electron Microscopy (Cryo-SEM), fluorescence analysis, and drug loading properties. In vitro studies of prepared LPs formulations were performed. The results revealed that LPs-loaded CDs-NHF significantly reduced mammary and lung cancer cell viability at lower doses compared to control CDs-NHF. That effect was further potentiated by LPs-CDs-NHF association with DOX. CDs-NHF-loaded LPs affected the expression of major molecules involved in oncogenesis like AKT, mTOR, and ERK.

Among various clinically used chemotherapeutic agents, doxorubicin (DOX) was selected in this study due to its extensive application in breast and lung cancer therapies, which represent the main focus of this investigation. Additionally, DOX is known for its compatibility with liposomal encapsulation—as evidenced by several approved liposomal formulations such as Doxil^®^—and possesses intrinsic fluorescence, which facilitates dual-tracking when combined with CDs-NHF. Furthermore, its amphiphilic nature allows for efficient entrapment within lipid bilayers, making it an ideal candidate for inclusion in a multifunctional liposomal delivery system with both therapeutic and diagnostic potential.

## 2. Results and Discussion

### 2.1. Preparation and Characterization of LPs Formulations

An important role in the preparation of LPs formulations is played by the selection of lipid composition which usually dictates the drug binding, distribution, and retention capacity of the LPs, respectively, and the drug release rate from the LPs. Also, another important parameter that can modulate membrane permeability and biological stability is cholesterol. This study aimed to examine the antitumoral activity of developed LPs loaded with CDs-NHF; DOX or CDs-NHF-DOX. LPs size, zeta potential, drug/lipid content, and the antitumoral effect using in vitro cancer cell line models were studied.

#### 2.1.1. LPs Formulations Preparation

The reverse-phase evaporation (REV) method was selected for liposome preparation due to its versatility and high encapsulation efficiency, especially for amphiphilic and hydrophilic molecules such as CDs-NHF and DOX. Unlike other methods, like thin-film hydration or ethanol injection, the REV method facilitates the formation of multilamellar vesicles (MLVs) with large internal aqueous volumes, providing an ideal microenvironment for the co-loading of therapeutic agents with different solubility profiles. Additionally, the process allows for better control over vesicle size and lamellarity, which is critical for the reproducibility and scale-up of multifunctional liposomal systems. Given the goal of integrating two bioactive compounds with distinct mechanisms of action into a stable nanosystem, REV proved to be the most appropriate and efficient approach.

The proposed pathway to obtain LPs based on Phospholipon 90G and cholesterol was a reverse-phase evaporation method, slightly modified [38]. The selected method involves the dissolution of the PC-G and CHOL in 10 mL of solvent (diethyl ether).

Next, a fixed volume of the previously prepared PBS or CDs-NHF/CDs-NHF-DOX/DOX suspension was slowly added dropwise to the solvent phase using a 1 mL syringe, through a rubber septum. During this step, the lipids spontaneously rearranged at the water/oil interface, leading to the formation of a microemulsion. The microemulsion was then subjected to sonication for 2 min to ensure the formation of a homogeneous dispersion.

Subsequently, the resulting microemulsion was transferred to a rotary evaporator, where the solvent phase was slowly removed under reduced pressure. The gradual removal of the solvent facilitated the formation of liposomes (LPs). The resulting LPs suspension was further homogenized using a vortex mixer for 2 min and placed in an ultrasonic bath for 30 min to obtain a uniform dispersion and dissociate any pre-formed aggregates. To reduce the size of the LPs, the final aqueous suspension was sequentially extruded through 400 nm, 200 nm, and 100 nm polycarbonate membranes using a mini-extruder (Avanti Polar Lipids, Alabaster, AL, USA). The obtained LPs were then purified by dialysis against distilled water for five days. The parameters used in the LP preparation process are summarized in Table 1.

Next, the LPs PC-G concentration (mg/mL) was determined via a Stewart assay and the results can be seen in Table 1. The variation in the PC-G concentration after the purification step was between 12 and 23 mg/mL. Moreover, the LPs’ potential for effective use as a controlled drug delivery system for different biomedical applications was analyzed. Considering the possibility of reducing the nonspecific organ toxicity, LPs were loaded with CDs-NHF, DOX, and CDs-NHF-DOX, respectively. The prepared liposomes were capable of including 98.2% of CDs-NHF and 97.7% of DOX, showing a high capacity of the LPs to entrap biologically active compounds.

DOX and CDs-NHF encapsulation efficiency (DEE) represents the drug quantity added (%) in the LPs preparation step and divided by the total drug quantity which is encapsulated in the LPs formulation. The DEE of DOX/CDs-NHF from LPs was calculated with Equation (1).

(1) $$DEE=\frac{Actual\;drug\left(DOX\;or\;CDs-NHF\right)\;content\;loaded\;in\;LPs}{Theoretical\;drug\;content\;(DOX)}\times 100$$

As shown in Table 1, the LP formulations exhibited a good encapsulation capacity. A higher drug concentration was observed for DOX (4.57 mg/mL) compared to CDs (2.27 mg/mL). However, when both CDs-NHF and DOX were co-loaded into the LPs, the overall concentration remained within a similar range.

#### 2.1.2. LPs Formulations Morphology

**Figure 1:**
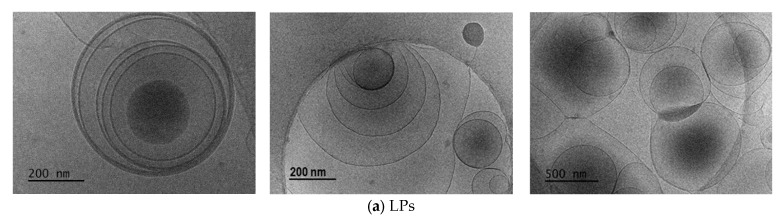

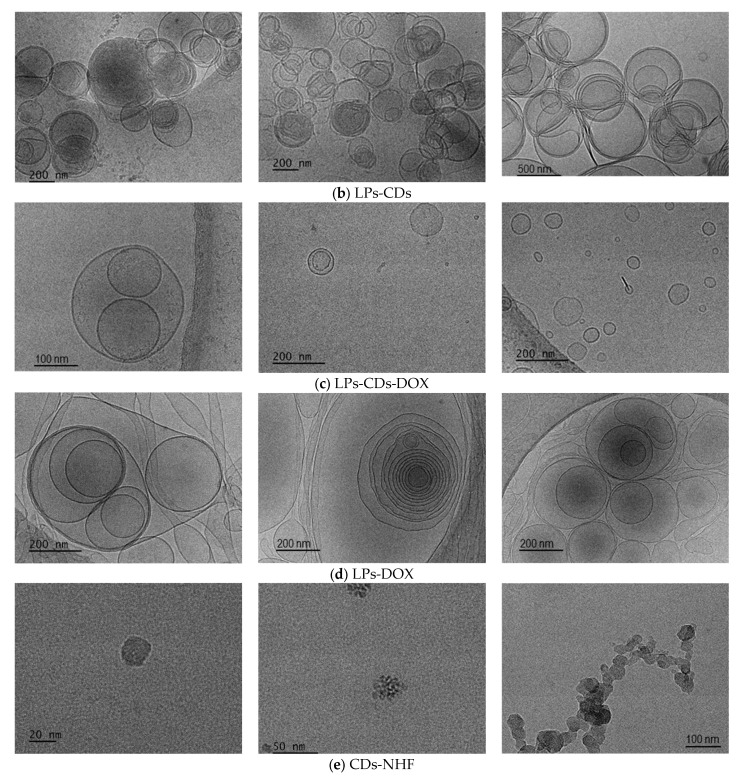
Cryo-TEM images for all LPs formulations and CDs-NHF. (**a**) Cryo-TEM images for LPs, (**b**) Cryo-TEM images for LPs-CDs, (**c**) Cryo-TEM images for LPs-CDs-DOX, (**d**) Cryo-TEM images for LPs-DOX, (**e**) Cryo-TEM images for the prepared CDs-NHF via the pyrolytic process revealed that obtained CDs-NHF are small entities in the 20–50 nm range.

**Figure 2:**
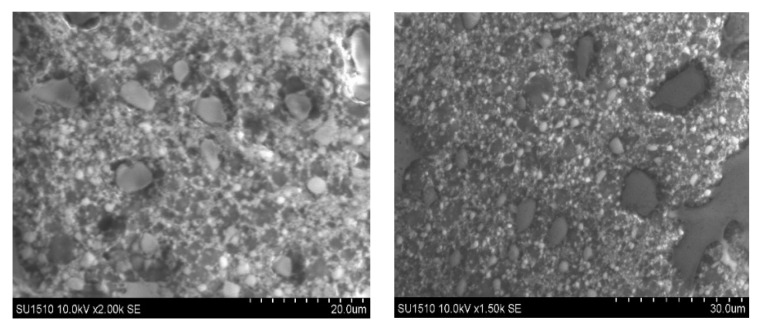
Cryo-SEM images of control LPs.

LPs morphology was investigated by Cryo-TEM (Figure 1) and Cryo-SEM (Figure 2), respectively.

Cryo-TEM micrographs of the unloaded liposomes, as well as those loaded with CDs-NHF, DOX, or both, confirmed the successful preparation of liposomal formulations as multilamellar vesicles (MLVs) with a spherical morphology (Figure 1). Also, the obtained MLVs are presenting several concentric bilayers with a mean particle size ranging from 300 to 1000 nm, in concordance with the DLS analysis results (Table 2, Figure S1). Cryo-TEM images of the LPs revealed discrete and intact structures without aggregation. A further investigation via Cryo-SEM (Figure 2) for control LPs also demonstrated that the LPs were obtained and presented a spherical shape.

#### 2.1.3. LPs Formulations Size and Zeta Potential

The size and zeta potential (ZP) of the liposomal formulations were evaluated, and the results are summarized in Table 2 and Supplementary Figure S1. The measurements revealed an average vesicle size ranging from 384 to 1000 nm. These findings confirm that the employed preparation method successfully yielded multilamellar vesicles (MLVs) with a characteristic spherical morphology, composed of multiple lipid bilayers encapsulating an aqueous core. Dynamic light scattering (DLS) analysis further indicated a size increase for LPs loaded with CDs-NHF, DOX, and CDs-NHF-DOX compared to the unloaded (control) liposomes. This enlargement in vesicle size may be attributed to interactions between the encapsulated CDs-NHF and/or DOX molecules and the lipid bilayers, potentially affecting the structural organization of the vesicles. Similar effects have been reported in the literature, where the incorporation of hydrophilic or amphiphilic agents leads to modifications in liposomal size and membrane dynamics due to drug–lipid interactions [39,40].

ZP knowledge of the prepared liposomal formulation is critical for the LPs design and quality control. LPs’ ZP was evaluated to establish surface charge stability via the Smoluchowski Equation (2):

(2) $$\xi =\frac{\eta µ}{\varepsilon }\;and\;k\alpha \gg 1$$

where

η—viscosity;
ε—dielectric constant;
k and α—Debye-Hűckel parameter, and LPs radius.

As a result, the LPs’ ZP (Supplementary Figure S2 and Table 2) revealed that MLVs formulations are presenting a high dispersion stability, which leads to a strong electrostatic repulsion interaction preventing the aggregation of MLVs and a higher entrapment capacity, respectively [40,41].

#### 2.1.4. LPs Formulations Fluorescence Abilities

**Figure 3:**
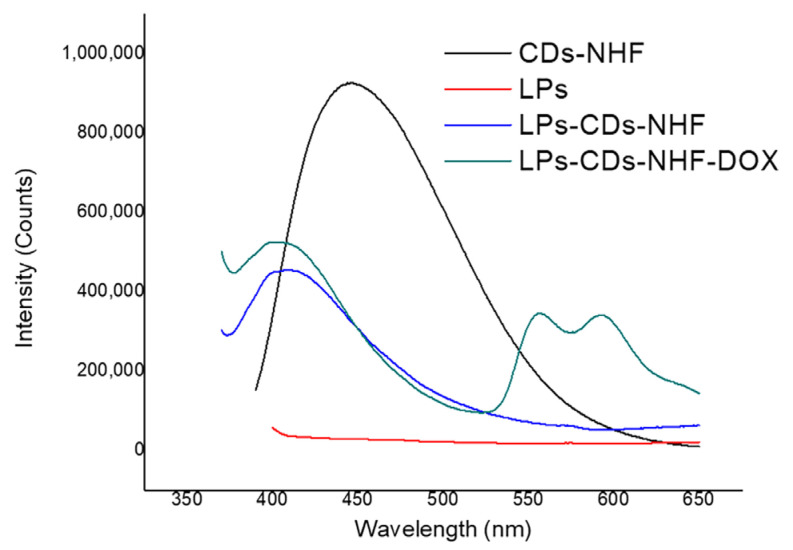
Emission spectra of CDs-NHF, LPs, LPs-CDs-NHF, and LPs-CDs-NHF-DOX formulations suspended in H_2_O.

Figure 3 and Table 3 present the emissions profiles of CDs-NHF, unloaded liposomes (LPs), LPs loaded with CDs-NHF, and LPs loaded with CDs-NHF-DOX, recorded at an excitation wavelength of 370 nm. The results show that the control LPs exhibit no detectable emissions, in contrast to the pronounced photoluminescence of CDs-NHF and the corresponding loaded LP formulations. For free CDs-NHF, a maximum emission intensity was observed at 447 nm. In comparison, the emission peaks of CDs-NHF in the LPs-CDs-NHF and LPs-CDs-NHF-DOX systems were shifted to 408 nm and 405 nm, respectively, (as shown in Table 3). Furthermore, the emission intensity of CDs-NHF was reduced by approximately 50% upon encapsulation within the liposomal structure. This decrease in emission intensity could be attributed to the interaction with the liposomal bilayer, which may partially quench or shield the fluorescent signal, potentially through the interference with the optical pathway or limited accessibility of the excitation/emission light. These observations suggest that the phosphatidylcholine-based lipid (PC-G) matrix plays a significant role in modulating the photoluminescence behavior of CDs-NHF within the LP formulations.

In addition, the photoluminescent properties of the LPs formulations were visually assessed under both daylight and UV illumination using a Philips UVA TL4WBLB lamp (emission maximum at 370–390 nm) and a 50 mW laser diode at 440 nm (Supplementary Figure S3). The visual observations were consistent with the fluorescence spectroscopy results, further confirming the presence and emission behavior of the CDs.

### 2.2. In Vitro Studies

The liposomal formulations containing CDs-NHF significantly inhibited the viability of breast (MDA-MB-231) and lung (A549) cancer cells. Given the high mortality and incidence associated with these types of cancer, we evaluated the effects of several formulations: empty liposomes (LPs), unencapsulated CDs-NHF, and LPs-CDs-NHF. All were tested at concentrations of 0.1%, 1%, and 5%.

**Figure 4:**
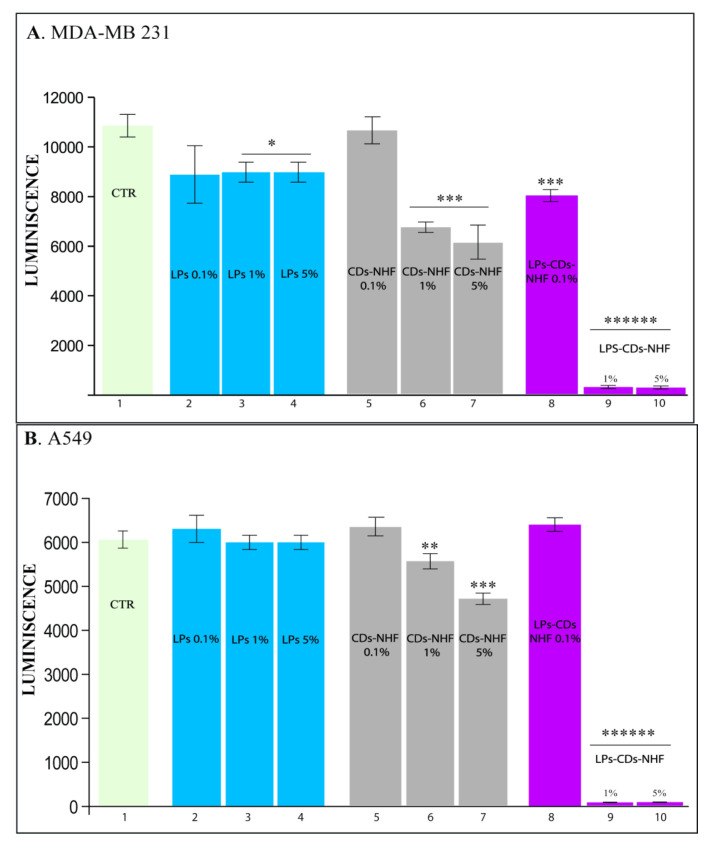
The effect of LPs-loaded CDs-NHF in cancer cell viability: (**A**) MDA-MDB231; (**B**) A549. 1 untreated; 2–4 empty LPs; 5–7 unencapsulated CDs-NHF; and 8–10 LPs encapsulated CDs-NHF. * *p* <0.05; ** *p* < 0.005; *** *p* < 0.0005; and ****** *p* < 0.00000005.

Figure 4 shows that CDs-NHF at a 5% concentration inhibited cancer cell proliferation, consistent with our previous findings [37]. Moreover, liposomal encapsulation of CDs-NHF amplified the antitumor effect, particularly in MDA-MB-231 and A549 cell lines (Figure 4A,B, columns 9–10). Notably, this treatment did not affect the viability of the normal breast epithelial cell line MCF-10A (Supplementary Figure S4).

**Figure 5:**
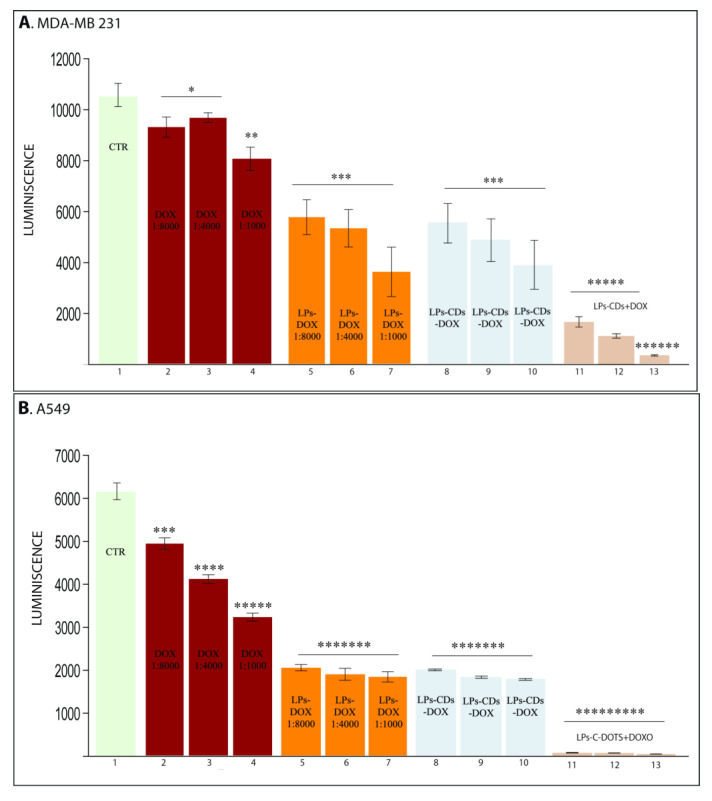
The effect of Doxorubicin in cancer cell viability: (**A**). MDA-MDB231; (**B**). A549. 1 untreated; 2–4 DOX; 5–7 LPs encapsulated DOX; 8–10 LPs encapsulated CDs-NHF—DOX (0.1%, 1%, and 5%/1:8000); and 11–13 LPs encapsulated CDs-NHF (0.1%, 1%, and 5%) suspended in DOX (1:8000). * *p* < 0.05; ** *p* < 0.005; *** *p* < 0.0005; **** *p* <0.00005; ***** *p* <0.000005; ****** *p* <0.0000005; ******* *p* < 0.00000005, and ********* *p* < 0.0000000005.

DOX-loaded liposomes also reduced breast and lung cancer cell viability. While DOX is a widely used chemotherapeutic agent, its clinical application is limited by systemic side effects [22]. LPs have been shown to enhance the therapeutic index of drugs, and this effect was also evident in our study (Figure 5). Four different formulations were tested: free DOX, DOX-loaded LPs, LPs containing both CDs-NHF and DOX, and LPs with CDs-NHF suspended in a DOX solution.

DOX significantly reduced cancer cell viability (Figure 5A,B, columns 2–4), and this effect was further enhanced by DOX-loaded liposomes (columns 5–7). Interestingly, LPs containing both CDs-NHF and DOX (columns 8–10) did not show further potentiation compared to DOX-LPs alone. However, when CDs-NHF-loaded LPs were administered alongside free DOX (columns 11–13), a marked reduction in cell viability was observed. This suggests that co-encapsulation may limit the efficacy of CDs-NHF, potentially due to interference within the same endocytic pathway, while separate administration allows each agent to exert its effect independently and synergistically.

CDs-NHF treatment also reduced the expression of phosphorylated Akt (pAkt 1/2/3), a key player in the PI3K/Akt/mTOR pathway implicated in tumor progression and treatment resistance [17,18]. The Akt family members—Akt 1,2,3—have different roles in invasion, migration, and metastasis dissemination [42].

**Figure 6:**
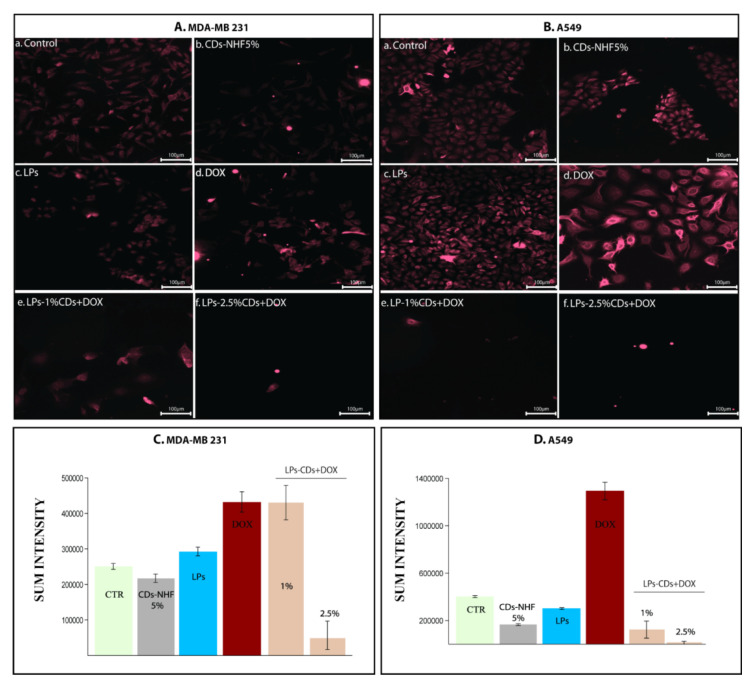
Level of pAkt (1,2,3): (**A**). MDA-MB231 cells; (**B**). A549 cells; (**Aa**,**Ba**) untreated; (**Ab**,**Bb**) unencapsulated CDs-NHF 5%; (**Ac**,**Bc**) LPs; (**Ad**,**Bd**) free DOX; (**Ae**,**Be**) LPs encapsulated CDs-NHF 1% suspended in DOX; (**Af**,**Bf**) LPs encapsulated CDs-NHF 2.5% suspended in DOX; and (**C**,**D**) quantification of fluorescence straining.

Figure 6 shows that 5% CDs-NHF significantly downregulated pAkt in both MDA-MB-231 and A549 cells (Figure 6Ab,Bb) compared to untreated controls (Aa, Ba). DOX treatment, on the other hand, increased pAkt expression and altered cell morphology, particularly in A549 cells (Ad, Bd). The combination of LPs-loaded CDs-NHF with free DOX further reduced the pAkt expression, except in the MDA-MB-231 group treated with 1% CDs-NHF, likely due to insufficient concentration to overcome treatment resistance. All the obtained data highlights the important ability of CDs-NHF encapsulated in LPs to reduce metabolic activities in investigated malignant cells.

To further investigate the underlying mechanisms, pmTOR expression was assessed and found to be reduced following CDs-NHF treatment. The mammalian target of rapamycin (mTOR), a downstream effector of Akt, plays a key role in cancer cell proliferation, apoptosis, and cell cycle regulation [43].

**Figure 7:**
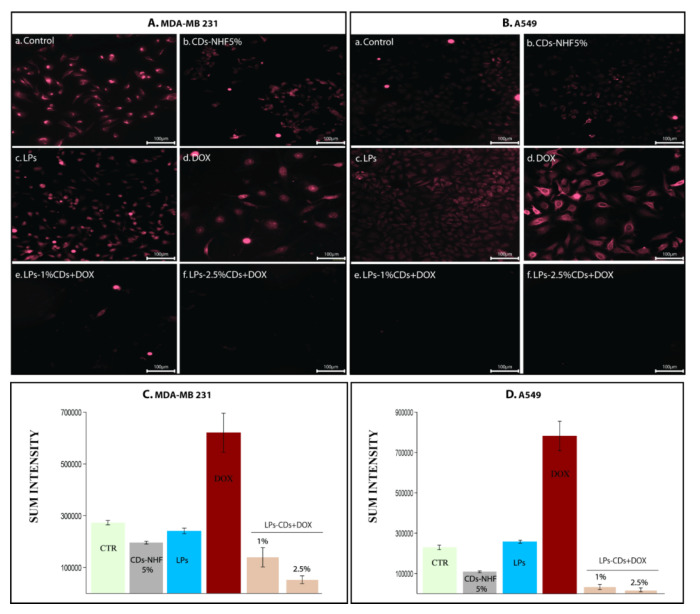
Expression levels of pmTOR: (**A**). MDA-MB231 cells; (**B**). A549cells; (**Aa**,**Ba**) untreated; (**Ab**,**Bb**) unencapsulated CDs-NHF 5%; (**Ac**,**Bc**) LPs; (**Ad**,**Bd**) free DOX; (**Ae**,**Be**) LPs encapsulated CDs-NHF 1% suspended in DOX; (**Af**,**Bf**) LPs encapsulated CDs-NHF 2.5% suspended in DOX; and (**C**,**D**) quantification of fluorescence straining.

Downstream of pAkt, pmTOR expression was also reduced following CDs-NHF treatment Figure 7Ab,Bb, whereas it increased in DOX-treated groups (Ad,Bd). Co-administration of LPs-loaded CDs-NHF and soluble DOX reduced pmTOR expression across all treatment groups (Ae–f,Be–f), highlighting the potential of this combination to inhibit the PI3K/Akt/mTOR signaling axis.

Treatment-dependent changes were also observed in the pERK1/2 expression. This protein, part of the MAPK signaling cascade, is involved in cancer cell migration and invasion [44].

**Figure 8:**
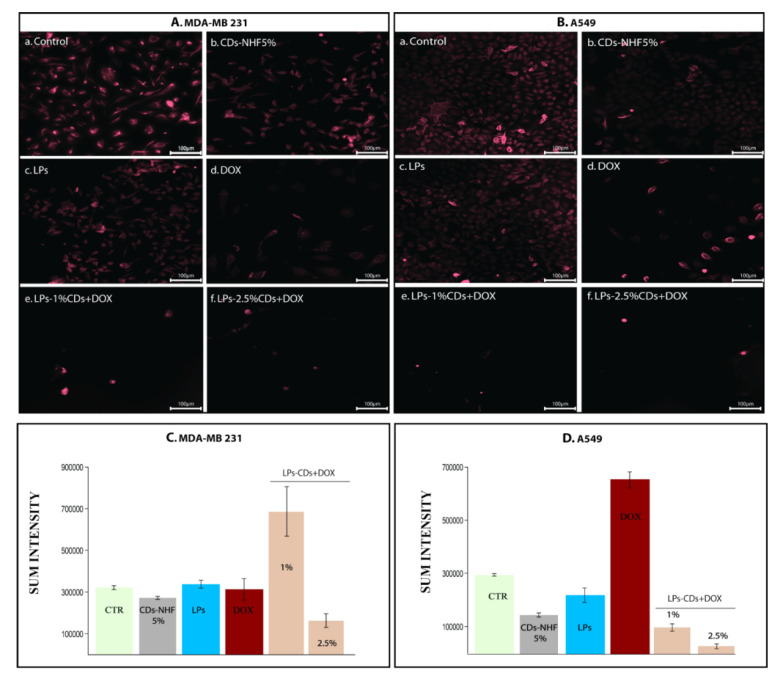
Level of pERK1/2: (**A**). MDA-MB231 cells; (**B**). A549 cells; (**Aa**,**Ba**) untreated; (**Ab**,**Bb**) unencapsulated CDs-NHF; (**Ac**,**Bc**) LPs; (**Ad**,**Bd**) free DOX; (**Ae**,**Be**) LPs encapsulated CDs-NHF 1% suspended in DOX; (**Af**,**Bf**) LPs encapsulated CDs-NHF 2.5% suspended in DOX; and (**C**,**D**) quantification of fluorescence straining.

Moreover, triple-negative breast cancer (TNBC) patients with a high ERK expression have a poorer survival rate than those with ERK with a low expression [45,46,47,48]. Both cell lines exhibited high baseline pERK1/2 levels, which were significantly reduced by CDs-NHF (Figure 8Ab,Bb). DOX treatment increased the pERK expression in A549 cells only (Figure 8Ad,Bd), indicating cell-type-specific responses. Notably, LPs-loaded CDs-NHF combined with DOX reduced pERK1/2 levels in both cell types, although 1% CDs-NHF showed an unexpected increase, again suggesting a concentration-dependent effect.

Our previous studies demonstrated that CDs-NHF reduce HSP90 expression in both in vivo and ex vivo cancer models, supporting their role in modulating oncogenic stress responses. Specifically, in a 4T1 murine breast cancer model, CDs-NHF treatment led to a decreased HSP90 and Ki67 expression as well as reduced metastatic dissemination, while in U87 glioma cells, CDs-NHF suppressed pAKT, p70S6Kinase, and other key signaling molecules involved in tumor survival and progression. These results suggest that the observed downregulation of HSP90 may be mediated indirectly through the inhibition of the PI3K/AKT axis and related pathways [45,46].

**Figure 9:**
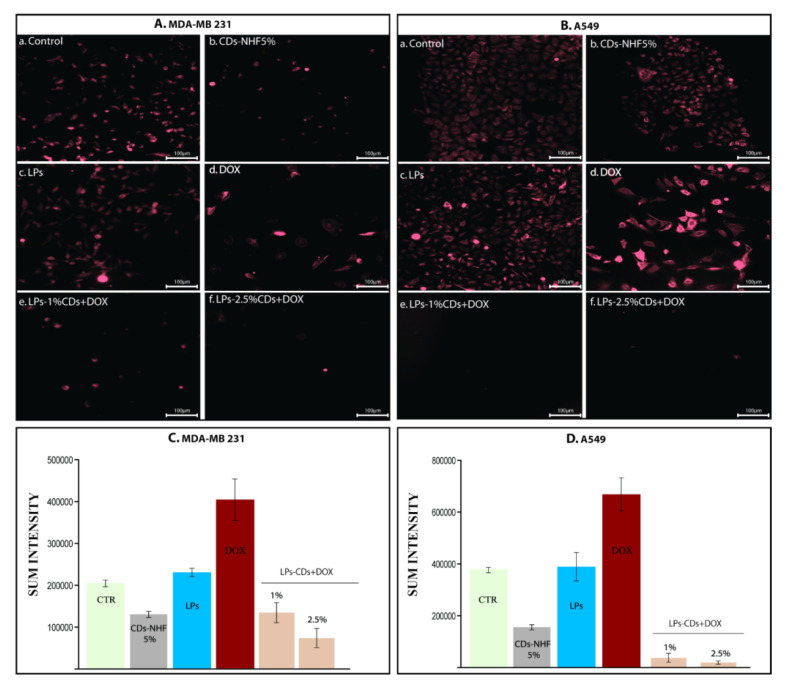
Expression levels of HSP90: (**A**). MDA-MB231 cells; (**B**). A549 cells; (**Aa**,**Ba**) untreated; (**Ab**,**Bb**) unencapsulated CDs-NHF; (**Ac**,**Bc**) LPs; (**Ad**,**Bd**) free DOX; (**Ae**,**Be**) LPs encapsulated CDs-NHF 1% suspended in DOX; (**Af**,**Bf**) LPs encapsulated CDs-NHF 2.5% suspended in DOX; and (**C**,**D**) quantification of fluorescence straining.

CDs-NHF treatment reduced HSP90 expression, a molecular chaperone involved in cancer progression (Figure 9). Combined treatment with encapsulated CDs-NHF and DOX further suppressed HSP90 levels, except in the group with 1% CDs-NHF, which was comparable to CDs-NHF alone. DOX treatment alone increased HSP90 expression.

The intrinsic antitumor activity of CDs-NHF is likely mediated through a combination of mechanisms. Prior in vivo and ex vivo studies have demonstrated that CDs-NHF induce oxidative stress and mitochondrial dysfunction, while also modulating key oncogenic signaling cascades such as PI3K/Akt and MAPK. These effects result in the downregulation of HSP90, Ki67, pAKT, p70S6K, and pERK, impairing tumor cell viability, proliferation, and invasiveness [45,46]. Notably, these effects appear to be selective for malignant cells, as no significant cytotoxicity was observed in normal epithelial cells, further supporting the therapeutic potential of CDs-NHF as a multifunctional antitumor agent.

Together, these results indicate that CDs-NHF impacts key oncogenic pathways (PI3K/Akt/mTOR and MAPK) and that combining CDs-NHF with DOX enhances this effect. Nanotechnology-based formulations, such as LPs, offer promising strategies to improve drug efficacy and reduce toxicity [29,47,48,49,50,51,52,53].

## 3. Materials and Methods

### 3.1. Materials

The following materials were used for the experiments: Phospholipon 90G (phosphatidylcholine, from Lipoid), cholesterol, N-hydroxyphthalimide, ferric chloride hexahydrate, ammonium thiocyanate, and diethyl ether were purchased from Sigma-Aldrich (St. Louis, MO, USA), doxorubicin (from Teva Pharmaceuticals Srl, Bucharest, Romania) and Milli-Q ultrapure distilled water from Merck Chemicals (Darmstadt, Germany). MCF-10A (ATCC) (a gift from Prof. Dr. J. Lorens) was maintained in DMEM/F12 supplemented with 5% horse serum; 20 ng/mL EGF; 10 ug/mL insulin; 0.5 ng/mL hydrocortisone; 10 ng/mL cholera toxin; 1% Pen/Strep; and bovine serum were purchased from Sigma-Aldrich (St. Louis, MO, USA). MDA-MB-231 (ATCC) was cultured in F-12K Medium, supplemented with 100 U/mL of penicillin and 100 μg/mL of streptomycin and 5% bovine serum; A549 (ATCC) was cultured in F-12K Medium, supplemented with 100 U/mL of penicillin and 100 μg/mL of streptomycin and 5% bovine serum. Mouse anti-human pAkt sc-271966, Mouse anti-human pmTOR sc-293133, Mouse anti-human pERK1/2 sc-13652, and HSP90 (Cell Signaling Technology, Bucharest, Romania, E289) were used for immunofluorescence staining. All reagents were used as provided, without further purification or modification.

### 3.2. Instrumentation

The morphology of LPs, CDs, LPs-CDs-NHF, LPs-DOX, and LPs-CDs-NHF-DOX was characterized by Cryogenic Transmission Electron Microscopy (Cryo-TEM) images, which were obtained using a Tecnai F20 X TWIN microscope (FEI Company, Hillsboro, OR, USA) equipped with a field emission gun, operating at an acceleration voltage of 200 kV. Images were recorded on the Gatan Rio 16 CMOS 4k camera (Gatan Inc., Pleasanton, CA, USA) and processed with Gatan Microscopy Suite (GMS) software (Gatan Inc., Pleasanton, CA, USA). Specimen preparation was performed by vitrification of the aqueous solutions on grids with holey carbon film (Quantifoil R 2/2; Quantifoil Micro Tools GmbH, Großlöbichau, Germany). Prior to use, the grids were activated for 15 s in oxygen plasma using a Femto plasma cleaner (Diener Electronic, Ebhausen, Germany). Cryo-samples were prepared by applying a droplet (3 μL) of the suspension to the grid, blotting with filter paper, and immediately freezing in liquid ethane using a fully automated blotting device Vitrobot Mark IV (Thermo Fisher Scientific, Waltham, MA, USA). After preparation, the vitrified specimens were kept under liquid nitrogen until they were inserted into a Cryo-TEM-holder Gatan 626 (Gatan Inc., Pleasanton, CA, USA) and analyzed in the TEM at −178 °C.

Also, LPs’ morphology was investigated by the Cryo-SEM technique. SEM images were recorded using a Hitachi SU 1510 (Hitachi Company, Japan) Scanning Electron Microscope, LPs were fixed on an aluminum stub and frozen at −20 °C with a Peltier stub before observation.

CDs-NHF, all LPs formulations, and cells mean diameter, size distribution, and zeta potential were analyzed via Delsa Nano C Submicron Particle Size Analyzer using the dynamic light diffusion (DLS), which measures the Brownian motion of the particles and correlates it with their size. The relationship between the size of a particle and its speed is given by the Stokes–Einstein Equation (3):

(3) $$DH=\frac{kBT}{3\pi \eta D}$$

where

DH is the hydrodynamic diameter;
D is the diffusion coefficient;
kB is Boltzmann’s constant;
T is the temperature;
η is the viscosity of the medium.

For sample preparation, 1–2 drops of each concentrated LPs formulation were dispersed in 2 mL of distilled water, followed by ultrasonication for 1 min. The zeta potential (ZP) analysis was conducted using the same protocol as the particle size measurements. All measurements were performed in triplicate to ensure reproducibility.

The photostability of CDs-NHF and all LPs formulations was assessed using a Horiba Fluoromax-4P spectrofluorometer (Fluor Essence software, Version 35.1.20) equipped with a Quanta-φ integrating sphere. Additionally, visual testing of photoluminescence properties was performed under a Philips UVA TL4WBLB lamp (München, Germany; λ_max = 370 nm) and a 50 mW laser diode emitting at 440 nm.

Cell viability studies were carried out using a multi-mode plate reader (FilterMax F5), while immunofluorescence imaging was performed on a Zeiss Axio Observer Z1 microscope (TissueGnostics platform, Zeiss, Hilden, Germany).

### 3.3. Methods

#### 3.3.1. Preparation of LPs Formulations

First, the carbon dots (CDs) were synthesized using a simple one-step pyrolytic method, as described by Stan et al. [54]. The resulting CDs exhibited a small average size of approximately 44 nm and were utilized in their suspension form for incorporation into the liposomal (LP) formulations.

To prepare the liposomal formulations—namely control liposomes (LPs), liposomes loaded with carbon dots (LPs-CDs-NHF), liposomes loaded with doxorubicin (LPs-DOX), and dual-loaded liposomes (LPs-CDs-NHF-DOX)—a slightly modified reverse-phase evaporation method, adapted from Xue et al. [38], was employed. Initially, Phospholipon 90G (phosphatidylcholine, PC-G; 60 mg) and cholesterol (CHOL; 40 mg) were dissolved in 10 mL of diethyl ether (in a 1:1.5 molar ratio) in a round-bottom flask.

For the preparation of control LPs, 1.5 mL of distilled water was added dropwise via a syringe through a rubber septum to form a water-in-oil emulsion. The mixture was then subjected to sonication for 2 min to facilitate emulsification. Subsequently, the solvent was removed by rotary evaporation (Heidolph Laborota 4002, 60 rpm, 35 °C, and under reduced pressure) until the disappearance of the gel phase was observed.

The resulting LPs suspension was homogenized by vortexing for 2 min and then placed in an ultrasound bath for 30 min to ensure uniform dispersion and to break potential aggregates. The liposomes were further size-reduced by sequential extrusion through polycarbonate membranes with pore sizes of 400 nm, 200 nm, and 100 nm using a mini-extruder (Avanti Polar Lipids, Alabaster, AL, USA).

For the preparation of LPs-CDs-NHF, LPs-DOX, and LPs-CDs-NHF-DOX, the same protocol was followed, with the exception that the 1.5 mL of distilled water was replaced with 1.5 mL of either an aqueous suspension of CDs-NHF (4 mg/mL), a DOX solution (2 mg/mL), or a combined CDs-NHF and DOX suspension.

Finally, all liposomal suspensions were purified by dialysis against Milli-Q water using cellulose ester membrane bags (molecular weight cutoff: 12,400 Da) for 5 days to remove unencapsulated components. The encapsulated amounts of carbon dots and/or doxorubicin were quantified by disrupting the liposomes with Triton X-100.

#### 3.3.2. Determination of Phospholipids Concentration by Stewart Assay

Phosphatidylcholine (PC) content was determined using the Stewart assay [55], a colorimetric method based on the formation of a red-colored complex between the phospholipid headgroups and ammonium ferrothiocyanate. This complex is extractable into an organic phase (chloroform) and can be quantitatively analyzed spectrophotometrically without interference from inorganic phosphate. All purified LPs formulations were analyzed using this technique.

The assay procedure included several key steps. Initially, a 0.1 M ammonium ferrothiocyanate solution was prepared, along with a standard calibration curve using known concentrations of PC-G. Standard solutions and six liposomal samples were transferred into 10 mL volumetric tubes, sealed with aluminum foil, and processed in triplicate. Each tube was vortexed vigorously for 15 s and left to stand at room temperature for 20 min to ensure complete reaction.

Following incubation, the aqueous phase (containing excess ammonium ferrothiocyanate) was carefully removed using a Pasteur pipette. The remaining organic (chloroform) phase, containing the PC-G complex, was analyzed at 485 nm using a UV-Vis spectrophotometer. The phosphatidylcholine concentration was calculated by comparing the absorbance values with the previously established calibration curve.

#### 3.3.3. In Vitro Studies

##### Cell Viability

Cell viability was assessed using the CellTiter-Glo^®^ Luminescent Cell Viability Assay. Cells were seeded in 96-well flat-bottom tissue culture plates at a density of 2000 cells/well and incubated overnight at 37 °C in a 5% CO_2_ atmosphere to allow adherence. After attachment, the cells were treated with different concentrations (0.1%, 1%, 2.5%, and 5%) of the following: empty liposomes (LPs), unencapsulated CDs-NHF, liposomes loaded with CDs-NHF, and liposomes loaded with CDs and suspended in DOX solution. Control cells received the same volume of medium used as a vehicle, as per the manufacturer’s instructions. After 72 h of treatment, 50 μL of the CellTiter-Glo^®^ reagent was added to each well, followed by 4 h of incubation. Luminescence was then measured using a multi-well plate reader (FilterMax F5).

##### Immunofluorescence (IF)

Samples were fixed with 4% paraformaldehyde for 30 min, then blocked in PBS containing 5% normal goat serum. Permeabilization was performed using 0.1% Triton X-100. Samples were incubated with the following primary antibodies for 72 h at 4 °C: anti-human pAkt (1:50), pmTOR (1:25), pERK44/42 (1:50), and HSP90 (1:25). After washing, cells were incubated with Alexa Fluor 647-conjugated secondary antibodies (1:200; Invitrogen). Images were acquired with a Zeiss Axio Observer Z1 microscope using TissueFaxs 4.2 software, and fluorescence intensity was quantified with TissueQuest 6.0.

##### Statistical Analysis

Statistical analyses were performed using GraphPad Prism, version Prism 8.0.0. The specific tests used are indicated in the figure legends. Grouped data were analyzed using one-way ANOVA, and statistical significance was considered at *p* < 0.05.

## 4. Conclusions

This study demonstrates the feasibility of liposome (LP) preparation using a slightly modified reverse-phase evaporation method. The resulting LPs formulations were characterized by Cryo-TEM and Cryo-SEM microscopy, which confirmed the formation of multilamellar vesicles (MLVs) with a spherical morphology. MLVs exhibited multiple concentric bilayers and had an average particle size ranging from 300 to 1000 nm. Furthermore, Cryo-TEM images revealed discrete, intact vesicles with no signs of aggregation, findings that were further corroborated by Cryo-SEM analysis. The drug loading capacity of the LPs, evaluated using CDs-NHF and/or free DOX, demonstrated high encapsulation efficiencies of up to 98.2% for CDs-NHF and 97.7% for DOX. The LPs formulations also exhibited good polydispersity and a negative zeta potential, indicating a high colloidal stability, which contributes to their enhanced drug entrapment capacity.

Finally, the antitumoral potential of CDs-NHF, DOX, and the co-loaded formulation (CDs-NHF-DOX) encapsulated within LPs was investigated, highlighting the promising application of these nano-formulations as drug delivery systems in biomedical contexts.

The in vitro study demonstrates that liposomes encapsulating CDs-NHF significantly reduce cancer cell viability in breast and lung cancer models compared to unencapsulated CDs-NHF. DOX-loaded liposomes were also more effective than free DOX. The combination of LPs-CDs-NHF with soluble DOX showed the highest cytotoxicity, likely due to synergistic effects. Mechanistically, CDs-NHF reduced the expression of pAkt (1,2,3), pmTOR, and pERK1/2, particularly when co-administered with DOX. HSP90 expression followed a similar trend. These findings support the use of CDs-NHF-loaded LPs, especially in combination therapies, as a promising nano-delivery platform for anticancer applications. Compared to conventional DOX–liposomal systems, the CDs-NHF/DOX co-loaded formulation presented here offers the added value of integrating a bioactive nanomaterial with proven antitumor potential, enabling a dual-action strategy that may enhance therapeutic efficacy while reducing DOX-associated toxicity.

## Figures and Tables

**Table 1:** LPs formulations preparation parameters.

Sample ID	Pre-Purification			Post-Purification	
	PC-G, mg/mL	CHOL, mg/mL	Diethyl Ether, mL	PC-G, mg/mL	CDs-NHF or DOX/Lipid, mg/mL
Control cells	-	-	-	-	-
Cells-loaded LPs	-	-	-	-	-
Cells-loaded LPs-CDs-NHF	-	-	-	-	-
CDs-NHF	-	-	-	-	-
Control LPs	60	40	10	23	-
LPs-CDs-NHF	60	40	10	17	0.13
LPs-DOX	60	40	10	13	0.35
LPs-CDs-NHF-DOX	60	40	10	12	0.219 (CDs)
					0.373 (DOX)

**Table 2:** Size distribution of LPs formulations.

Sample ID	Diameter (DLS ± SD), nm	Zeta Potential (mV)
Control cells	-	−8.11
Cells-loaded LPs	-	−6.10
Cells-loaded LPs-CDs-NHF	-	−11.98
CDs-NHF	44	−10.47
LPs	384	−33.12
LPs-CDs-NHF	1016	−24.86
LPs-DOX	917	−23.67
LPs-CDs-NHF-DOX	801	−26.57

**Table 3:** Fluorescence results obtained for CDs-NHF, LPs, LPs-CDs-NHF, and LPs-CDs-NHF-DOX formulations.

Sample ID	Excitation (nm)	Emission Peaks (nm)	Intensity (Counts)
CDs-NHF	370	447	9.27641 × 10^5^
Control LPs	370	0	0
LPs-CDs-NHF	370	408	4.55403 × 10^5^
LPs-CDs-NHF-DOX	370	405	5.26137 × 10^5^(CDs-NHF)
LPs-CDs-NHF-DOX	370	557	3.46898 × 10^5^(DOX)
LPs-CDs-NHF-DOX	370	593	3.42876 × 10^5^(DOX)

## Data Availability

All the data are in the manuscript and Supplementary Materials we provided.

## Supplementary Materials

The following are available online at https://www.mdpi.com/article/10.3390/ijms26157535/s1.
