# Supplementary material for: Carbon Dot-Enhanced Doxorubicin Liposomes: A Dual-Functional Nanoplatform for Cancer Therapy
Source: Int J Mol Sci. 2025 Aug 4;26(15):7535. doi: 10.3390/ijms26157535 (PMC12347740; doi:10.3390/ijms26157535)
Supplement: Supplementary file 1 [file ijms-26-07535-s001.zip › Supplementary Materials.pdf]

Supplementary Materials

Carbon Dot-Enhanced Doxorubicin Liposomes: A Dual-Functional Nanoplatform for Cancer Therapy

Corina-Lenuta Logigan [1,†], Cristian Peptu [2,†], Corneliu S. Stan [1], Gabriel Luta [3], Crina Elena Tiron [3,‡], Mariana Pinteala [2], Aleksander Foryś [4], Bogdan Simionescu [1,2,‡], Constanta Ibanescu [1], Adrian Tiron [3,*] and Catalina A. Peptu [1,*]

[1] Department of Natural and Synthetic Polymers, Faculty of Chemical Engineering and Environmental Protection, "Gheorghe Asachi" Technical University of Iasi, 700050 Iasi, Romania
[2] "Petru Poni" Institute of Macromolecular Chemistry, 700487 Iasi, Romania
[3] Regional Institute of Oncology, 700483 Iasi, Romania
[4] Centre of Polymer and Carbon Materials of the Polish Academy of Sciences, 41-819 Zabrze, Poland
* Correspondence: adrian.tiron@iroiasi.ro (A.T.); catalina-anisoara.peptu@academic.tuiasi.ro (C.A.P.); Tel.: +40-765-677-151 (A.T.); +40-765-253-915 (C.A.P.)
† These authors contributed equally to this work.
‡ Passed away.

Contents

**1.**	Intensity Distribution curves of a) control tumoral cells, b) cells with LPs; c) tumoral cells with LPs-CDs-NHF; d) CDs-NHF; e) Control LPs; f) LPs-CDs-NHF; g) LPs-DOX and h) LPs-CDs-DOX	***Figure S1***	*Page 2-3*
**2.**	Zeta Potential of a) control tumoral cells, b) cells with LPs; c) tumoral cells with LPs-CDs-NHF; d) CDs-NHF; e) Control LPs; f) LPs-CDs-NHF; g) LPs-DOX and h) LPs-CDs-DOX	***Figure S2***	*Page 4-6*
**3.**	Photographs of 1) CDs-NHF; 2) DOX; 3) Control LPs; 4) LPs-CDs-NHF; 5) LPs-DOX and 6) LPs-CDs-NHF-DOX formulation's under a) daylight and b) UV illumination	***Figure S3***	*Page 7*
**4.**	Viability of normal mammary cell line	***Figure S4***	*Page 7*

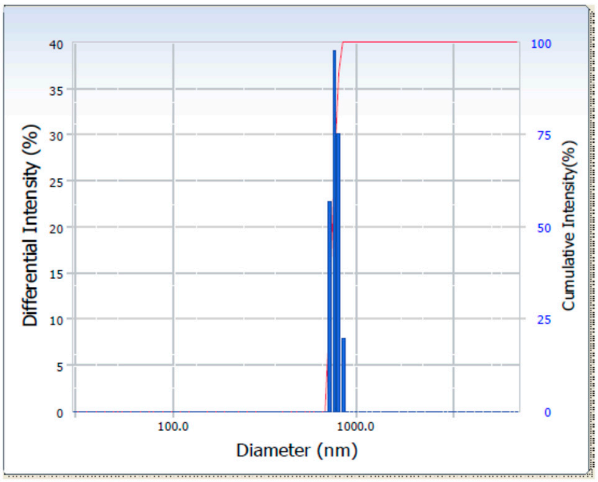

Differential Intensity (%)
Cumulative Intensity(%)
Diameter (nm)
0
5
10
15
20
25
30
35
40
100.0
1000.0
25
50
75
100

a)

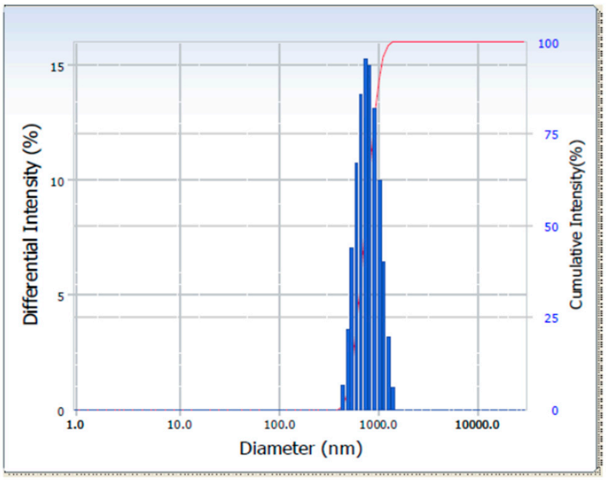

Differential Intensity (%)
Cumulative Intensity(%)
Diameter (nm)
0
5
10
15
1.0
10.0
100.0
1000.0
10000.0
25
50
75
100

b)

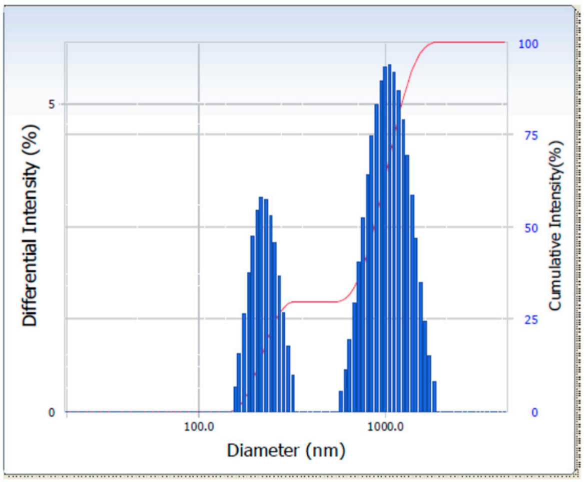

Differential Intensity (%)
Cumulative Intensity(%)
Diameter (nm)
0
5
100.0
1000.0
25
50
75
100

c)

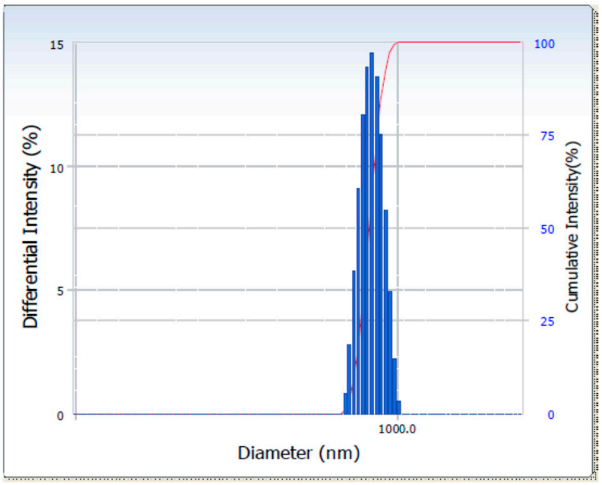

Differential Intensity (%)
Cumulative Intensity(%)
Diameter (nm)
0
5
10
15
1000.0
25
50
75
100

d)

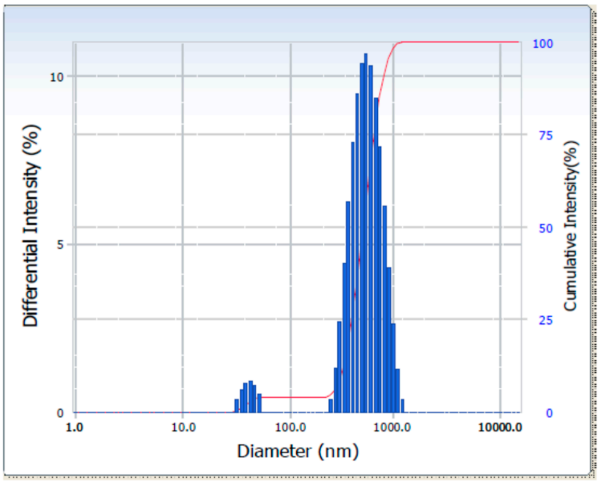

Differential Intensity (%)
Cumulative Intensity(%)
Diameter (nm)
0
5
10
1.0
10.0
100.0
1000.0
10000.0
25
50
75
100

e)

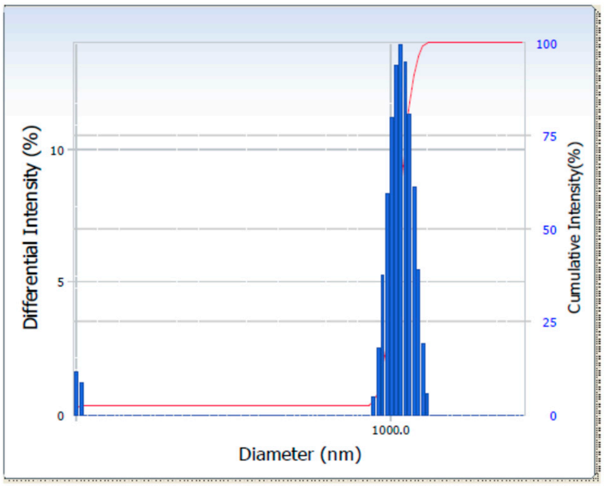

Differential Intensity (%)
Cumulative Intensity(%)
Diameter (nm)
0
5
10
1000.0
25
50
75
100

f)

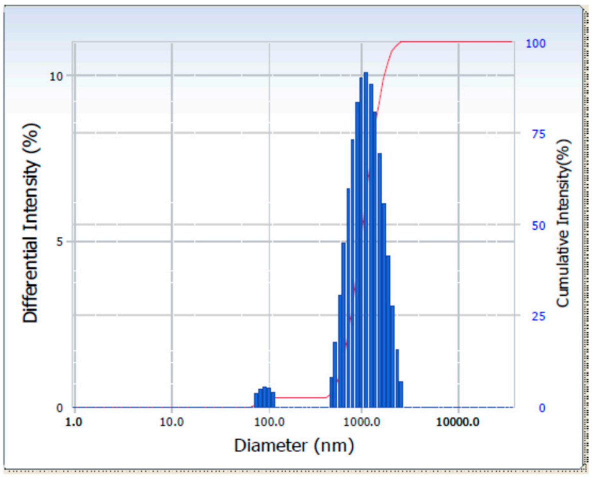

g)

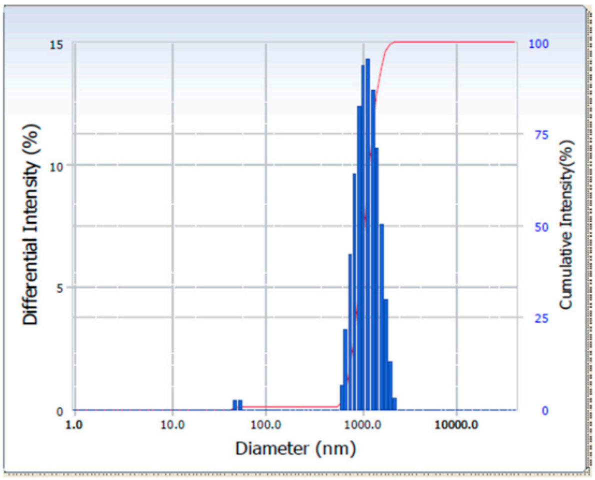

h)

Figure S1. Intensity Distribution curves of a) control tumoral cells, b) cells with LPs; c) tumoral cells with LPs-CDs-NHF; d) CDs-NHF; e) Control LPs; f) LPs-CDs-NHF; g) LPs-DOX and h) LPs-CDs-DOX

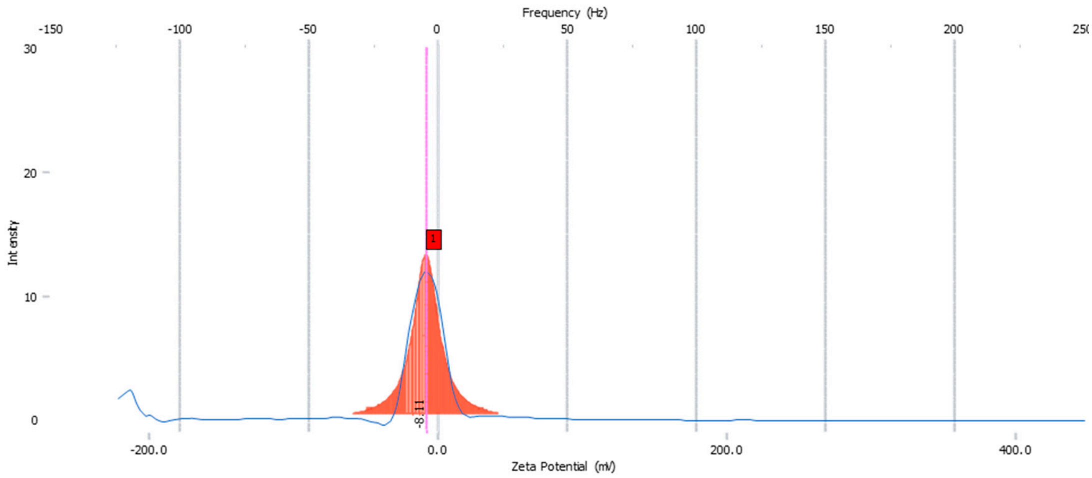
Frequency (Hz)
-150
-100
-50
0
50
100
150
200
250
30
20
10
0
Intensity
-8.11
1
-200.0
0.0
200.0
400.0
Zeta Potential (mV)

a)

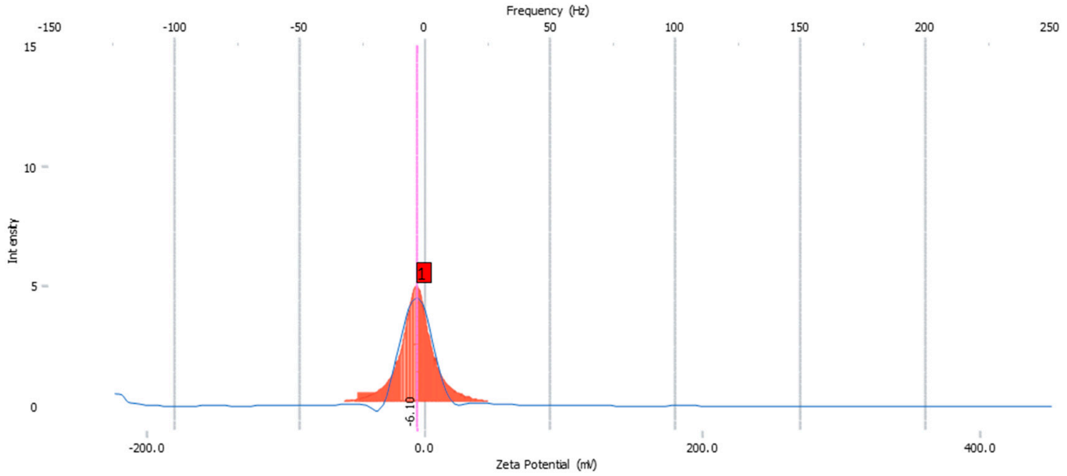
Frequency (Hz)
-150
-100
-50
0
50
100
150
200
250
15
10
5
0
Intensity
-6.10
1
-200.0
0.0
200.0
400.0
Zeta Potential (mV)

b)

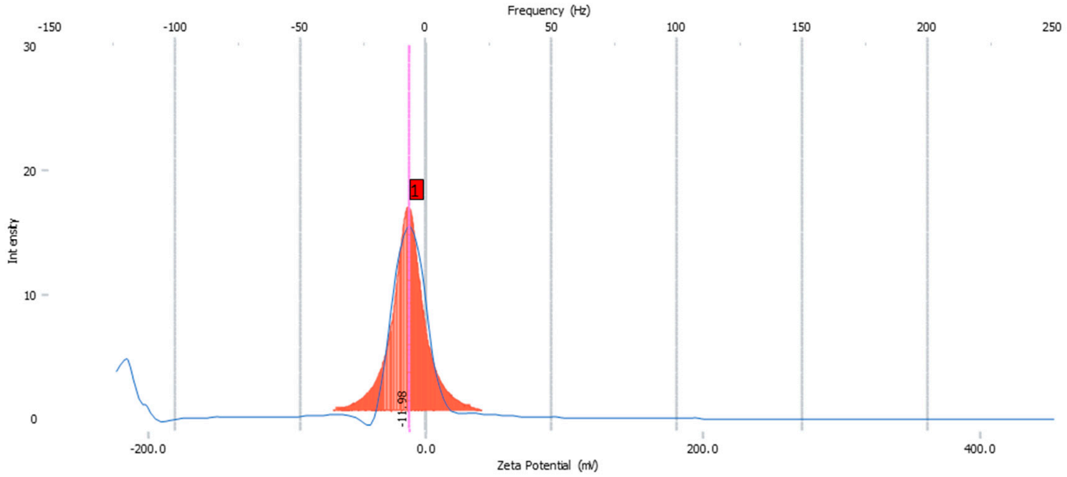
Frequency (Hz)
-150
-100
-50
0
50
100
150
200
250
30
20
10
0
Intensity
-11.98
1
-200.0
0.0
200.0
400.0
Zeta Potential (mV)

c)

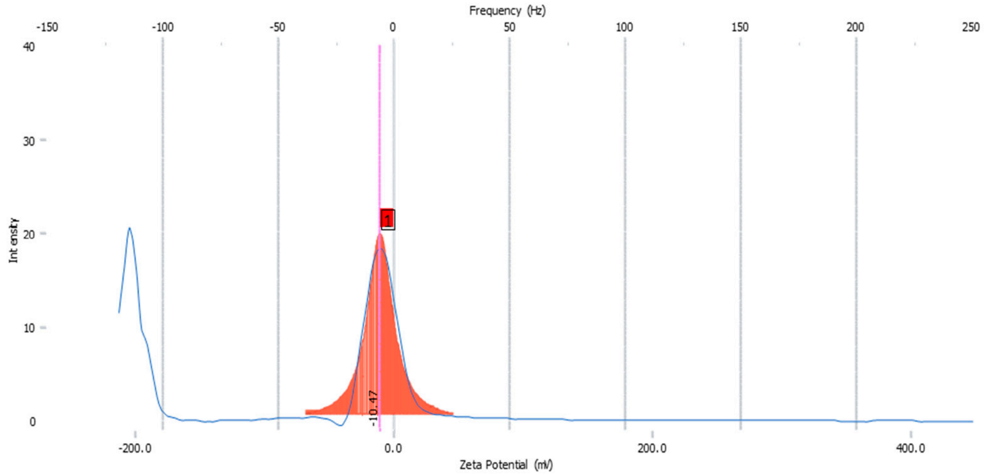
Frequency (Hz)
-150
-100
-50
0
50
100
150
200
250
40
30
20
10
0
Intensity
1
-10.47
-200.0
0.0
200.0
400.0
Zeta Potential (mV)

d)

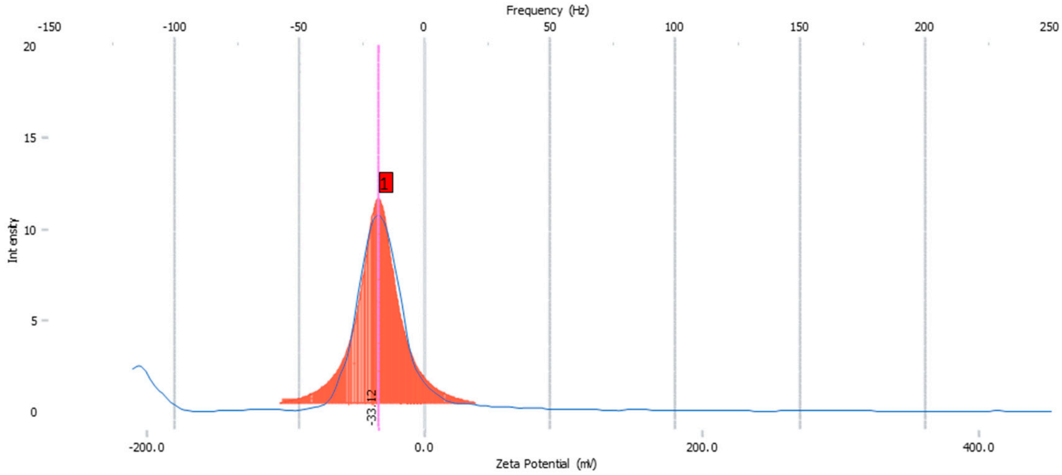
Frequency (Hz)
-150
-100
-50
0
50
100
150
200
250
20
15
10
5
0
Intensity
1
-33.12
-200.0
0.0
200.0
400.0
Zeta Potential (mV)

e)

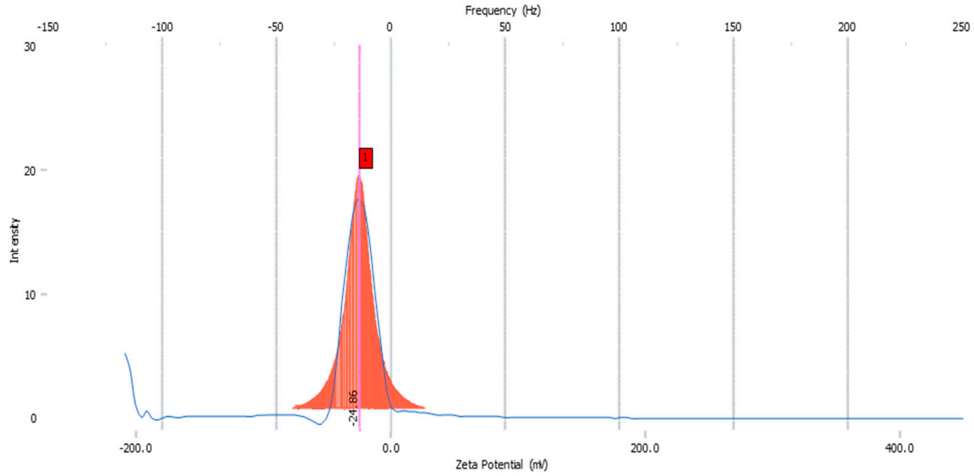
Frequency (Hz)
-150
-100
-50
0
50
100
150
200
250
30
20
10
0
Intensity
1
-24.86
-200.0
0.0
200.0
400.0
Zeta Potential (mV)

f)

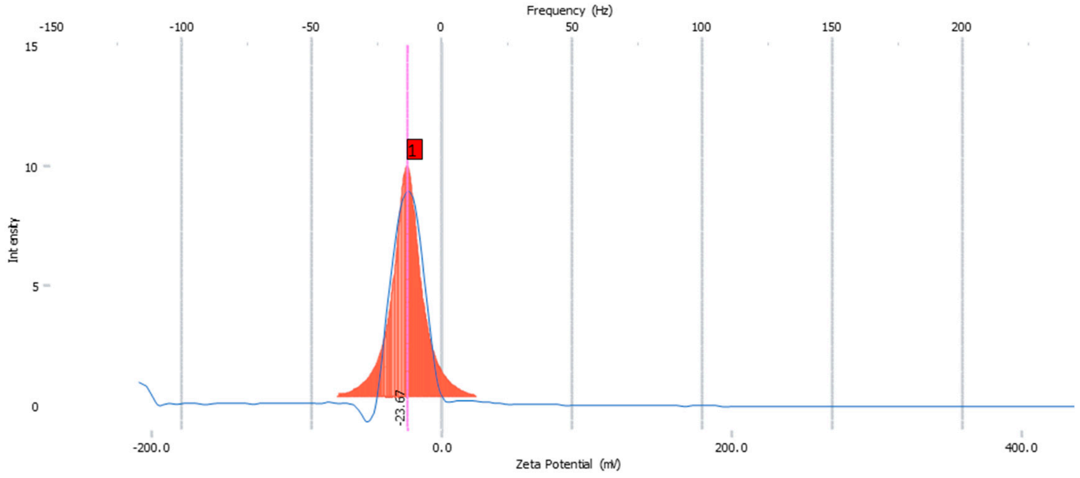

g)

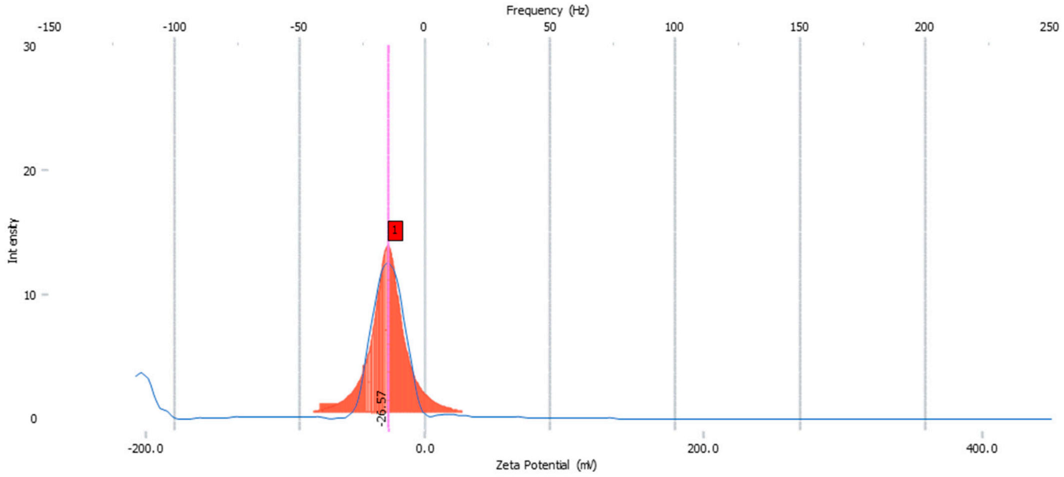

h)

Figure S2. Zeta Potential of a) control tumoral cells, b) cells with LPs; c) tumoral cells with LPs-CDs-NHF; d) CDs-NHF; e) LPs; f) LPs-CDs-NHF; g) LPs-DOX and h) LPs-CDs-DOX

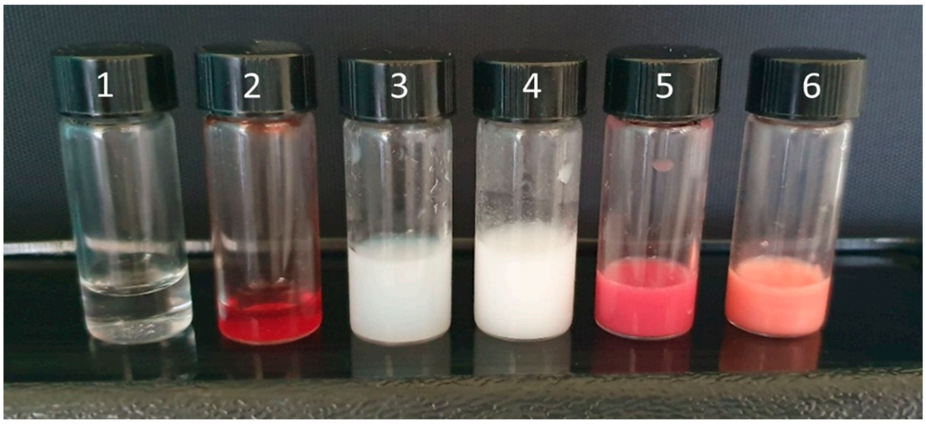

a)

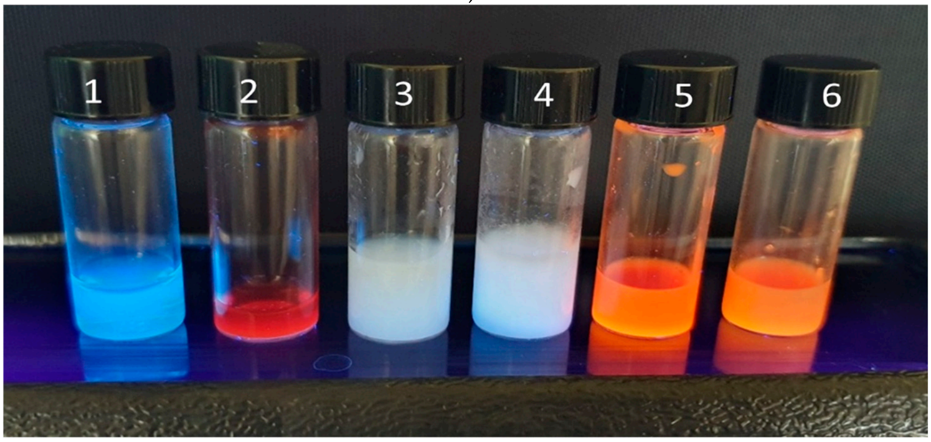

b)

Figure S3. Photographs of 1) CDs-NHF; 2) DOX; 3) Control LPs; 4) LPs-CDs-NHF; 5) LPs-DOX and 6) LPs-CDs-NHF-DOX formulations under a) daylight and b) UV illumination

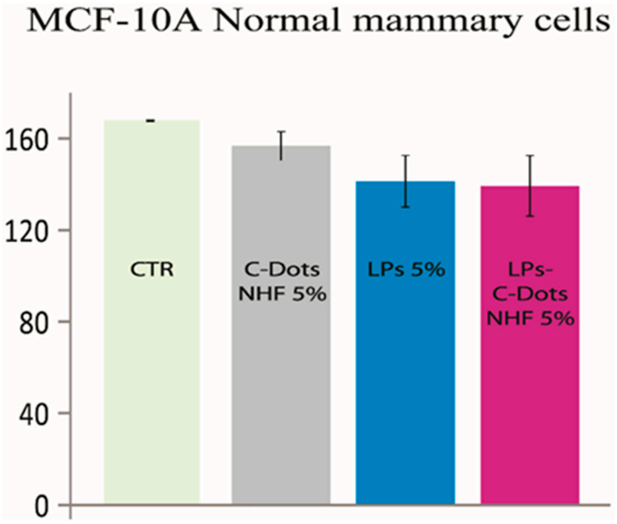

Figure S4. Viability of normal mammary cell line
